# Uncertainty analysis in internal dose calculations for cerium considering the uncertainties of biokinetic parameters and *S* values

**DOI:** 10.1007/s00411-020-00872-9

**Published:** 2020-09-20

**Authors:** Vladimir Spielmann, Wei Bo Li, Maria Zankl, Juan Camilo Ocampo Ramos, Nina Petoussi-Henss

**Affiliations:** 1 Institute of Radiation Medicine, Helmholtz Zentrum München-German Research Center for Environmental Health, Neuherberg, Germany; 2 CRCT, UMR 1037, INSERM, Université Toulouse III Paul Sabatier, Toulouse, France

**Keywords:** Uncertainty quantification, Internal dosimetry, Biokinetic model, Reference and non-reference phantoms

## Abstract

Radioactive cerium and other lanthanides can be transported through the aquatic system into foodstuffs and then be incorporated by humans. Information on the uncertainty of reported dose coefficients for exposed members of the public is then needed for risk analysis. In this study, uncertainties of dose coefficients due to the ingestion of the radionuclides ^141^Ce and ^144^Ce were estimated. According to the schema of internal dose calculation, a general statistical method based on the propagation of uncertainty was developed. The method takes into account the uncertainties contributed by the biokinetic models and by the so-called *S* values. These S-values were derived by using Monte Carlo radiation transport simulations with five adult non-reference voxel computational phantoms that have been developed at Helmholtz Zentrum München, Germany. Random and Latin hypercube sampling techniques were applied to sample parameters of biokinetic models and S values. The uncertainty factors, expressed as the square root of the 97.5th and 2.5th percentile ratios, for organ equivalent dose coefficients of ^141^Ce were found to be in the range of 1.2–5.1 and for ^144^Ce in the range of 1.2–7.4. The uncertainty factor of the detriment-weighted dose coefficient for ^141^Ce is 2.5 and for ^144^Ce 3.9. It is concluded that a general statistical method for calculating the uncertainty of dose coefficients was developed and applied to the lanthanide cerium. The dose uncertainties obtained provide improved dose coefficients for radiation risk analysis of humans. Furthermore, these uncertainties can be used to identify those parameters most important in internal dose calculations by applying sensitivity analyses.

## Introduction

As a result of human technical activities, the man-made and radioactive isotopes ^141^Ce and ^144^Ce might pollute the environment. Through different transport pathways, they could be spread and enter the human food chain via aquatic ecosystems (Kartha et al. 1998, U.S. EPA 2009, 2012). Determination of the internal dose after their ingestion is necessary to judge if any protective actions from exposure to these radionuclides are required for members of the public.

The International Commission of Radiological Protection (ICRP) recommends the following three quantities for use in radiological protection: the absorbed dose in an organ or tissue (organ absorbed dose), *D*_T_, the equivalent dose in an organ or tissue (organ equivalent dose), *H*_T_, and the effective dose, *E* (ICRP 1991, 2007). Absorbed, equivalent and effective doses to human tissue are not measurable but can be calculated if the corresponding exposure conditions are known. The coefficients (dose per administered activity) of these quantities from incorporated radionuclides can be calculated according to an approach recommended by the ICRP (ICRP 2015). In this schema, the calculation of dose coefficients depends on two models: the biokinetic and the dosimetric model. The biokinetic model (ICRP 1989, 2015) provides time-dependent activity curves of incorporated radionuclides in organs or tissue, while the dosimetric model (ICRP 2016) provides the specific fraction of energy of a type of radiation emitted within the source regions (e.g., organs) that is absorbed in the target regions. This so-called specific absorbed fraction (SAF) is calculated using Monte Carlo radiation transport simulation codes together with human voxel phantoms. The dosimetric model quantifies the transport of radiation in the body and therefore depends not only on radiation physics but also on the anatomy and geometrical organ features. Because of the uncertainties in the biokinetic and dosimetric parameters, the resulting equivalent and effective dose coefficients involve some uncertainties (NCRP 2009).

Dose coefficients after ingestion and inhalation of radionuclides by members of the public are reported by ICRP (ICRP 1989, 1993, 1995a; b, c) and revisions are underway, implementing the most recent ICRP dosimetric framework of internal dosimetry (ICRP 2007, 2009, 2016, 2020). The ICRP values are valid for the reference individual, obtained for simplified reference conditions, and are given without associated uncertainty. The lack of accuracy in radiation dose models varies for the various parameters and the circumstances in defined situations. Therefore, it is not possible to provide values for the uncertainties across the range of ICRP models, despite the fact that their assessment is an important part of model development. The ICRP is aware of the uncertainty in radiation dose models, and efforts are undertaken to critically evaluate and reduce them wherever possible. For regulatory purposes, the dosimetric models and parameter values that ICRP recommends are reference models and values. They are fixed by convention and therefore not subject to uncertainty (ICRP 2007). However, for special cases, uncertainties can be very important to judge the reliability and the potential range of the dose and risk estimates for real individuals and situations. A quantitative analysis of internal dose can help to guide regulators in setting appropriate limits on intakes (Görtz et al. 2000), to guide national and international organizations to invest scientific efforts for updating the models and dose coefficients (Bailey et al. 2007), and to provide dose uncertainties for occupational workers (Henrichs 2007). Quantification of uncertainties can support the reliability of dose coefficients as regulatory dose limits and constraints (Puncher et al. 2012). Furthermore, it is important for precise risk analysis (UNSCEAR 2012; NCRP 2014).

In the early time, Leggett and Williams (1981) defined a reliability index to measure the reliability of models. Later, the U.S. Nuclear Regulation Commission (NRC) Probabilistic Risk Analysis (PRA) Working Group has investigated uncertainties in internal dose assessment (Goossens et al. 1998). ICRP has published a series of papers where the reliability of the biokinetic models and the resulting dose coefficients for members of the public are discussed (Leggett et al. 1998, 2007; Harrison et al. 2001; Leggett 2001, 2003). The National Council on Radiation Protection and Measurement (NCRP) reported methods applicable to quantification on uncertainties in internal dose assessment (NCRP 2009). Based on biokinetic experimental investigations and developed voxel phantoms, the research group of the authors of this article has reported results of a series of studies on uncertainty analysis in internal dosimetry (Li et al. 2008, 2009, 2010, 2011, 2015; Schmidl et al. 2012; Höllriegl et al. 2020; Spielmann et al. 2016, 2018).

In the present study, a statistical method was developed to analyze the two sources of uncertainties in the calculation of dose coefficients, namely, the biokinetic model parameters, and the dosimetric parameters, the so-called *S* values, derived from different voxel phantoms and Monte Carlo radiation transport calculations.

Although many different phantoms have been developed around the world, *S* values are not completely calculated for all radionuclides and organs or are not always accessible. Therefore, a method was developed to consider the uncertainty of *S* values if available, and a case study was examined here with existing *S* values using phantoms that have been constructed at Helmholtz Zentrum München, Germany.

This method was applied to calculate the uncertainty of internal doses due to the incorporation of the radionuclides ^141^Ce and ^144^Ce. To quantify the uncertainty, the uncertainty factor UF, defined as the square root of the ratio between 97.5th and 2.5th percentiles, was estimated.

## Materials and methods

### Radionuclides

In the present study, the uncertainties of dose coefficients were calculated for ingestion of the cerium isotopes ^141^Ce [$T_{1/2} =$ 32.5 days, $\beta_{{{\text{av}}}}^{ - } =$ 129 keV (70%), $\beta_{{{\text{av}}}}^{ - } =$ 180 keV (30%), $\gamma =$ 145 keV (48%)], and ^144^Ce [$T_{1/2} =$ 284.9 days, $\beta_{{{\text{av}}}}^{ - } =$ 50 keV (19.2%), $\beta_{{{\text{av}}}}^{ - } =$ 66 keV (3.9%), $\beta_{{{\text{av}}}}^{ - } =$ 91 keV (76.9%), $\gamma$ = 41 keV (0.32%), 80 keV (1.4%), 134 keV (10.83%)]. The percentages in parentheses show the number of electrons and photons emitted per 100 disintegrations.

### Computation of organ and effective dose coefficients

Internal doses have been calculated following approach dosimetry published by the ICRP (2007, 2015).

1 $$ H\left( {r_{{\text{T}}} ,\tau } \right) = \mathop \sum \limits_{i} \mathop \sum \limits_{{r_{{\text{S}}} }} \tilde{A}\left( {r_{{\text{S}}} ,\tau } \right)S_{{\text{w}}} \left( {r_{{\text{T}}} \leftarrow r_{{\text{S}}} } \right)_{i} , $$

where $\tilde{A}\left( {r_{{\text{S}}} ,\tau } \right)$ is the time-integrated activity (or total number of nuclear transformations) in a source organ or region $r_{{\text{S}}}$ over the integration period $\tau$, $\tau$ is commonly taken to be 50 years, and $S\left( {r_{{\text{T}}} \leftarrow r_{{\text{S}}} } \right)$ is the radionuclide-specific quantity representing the mean weighted equivalent dose in target tissue $r_{{\text{T}}}$ due to nuclear transformations of radioisotope $i$ in source region $r_{{\text{S}}}$, the so-called *S* value.

Normalization to a unit administered activity $A_{0}$ provides equivalent dose coefficients $h\left( {r_{{\text{T}}} ,\tau } \right)$ in target tissue $r_{{\text{T}}}$.

The effective dose coefficient $e\left( \tau \right)$ for a reference person can be calculated, as defined by ICRP (2007, 2015), as a weighted sum of tissue-equivalent dose of the reference male and female according to the following formula:

2 $$ e\left( \tau \right) = \mathop \sum \limits_{T} w_{{\text{T}}} \left[ {\frac{{h\left( {r_{{\text{T}}} ,\tau } \right)^{{{\text{Male}}}} + h\left( {r_{{\text{T}}} ,\tau } \right)^{{{\text{Female}}}} }}{2}} \right], $$

where $w_{{\text{T}}}$ is the tissue-weighting factor for the target tissue $r_{{\text{T}}}$, and $h\left( {r_{{\text{T}}} ,\tau } \right)^{{{\text{Male}}}}$ and $h\left( {r_{{\text{T}}} ,\tau } \right)^{{{\text{Female}}}}$ are the equivalent dose coefficients for the male and female, respectively.

**Fig. 1:**
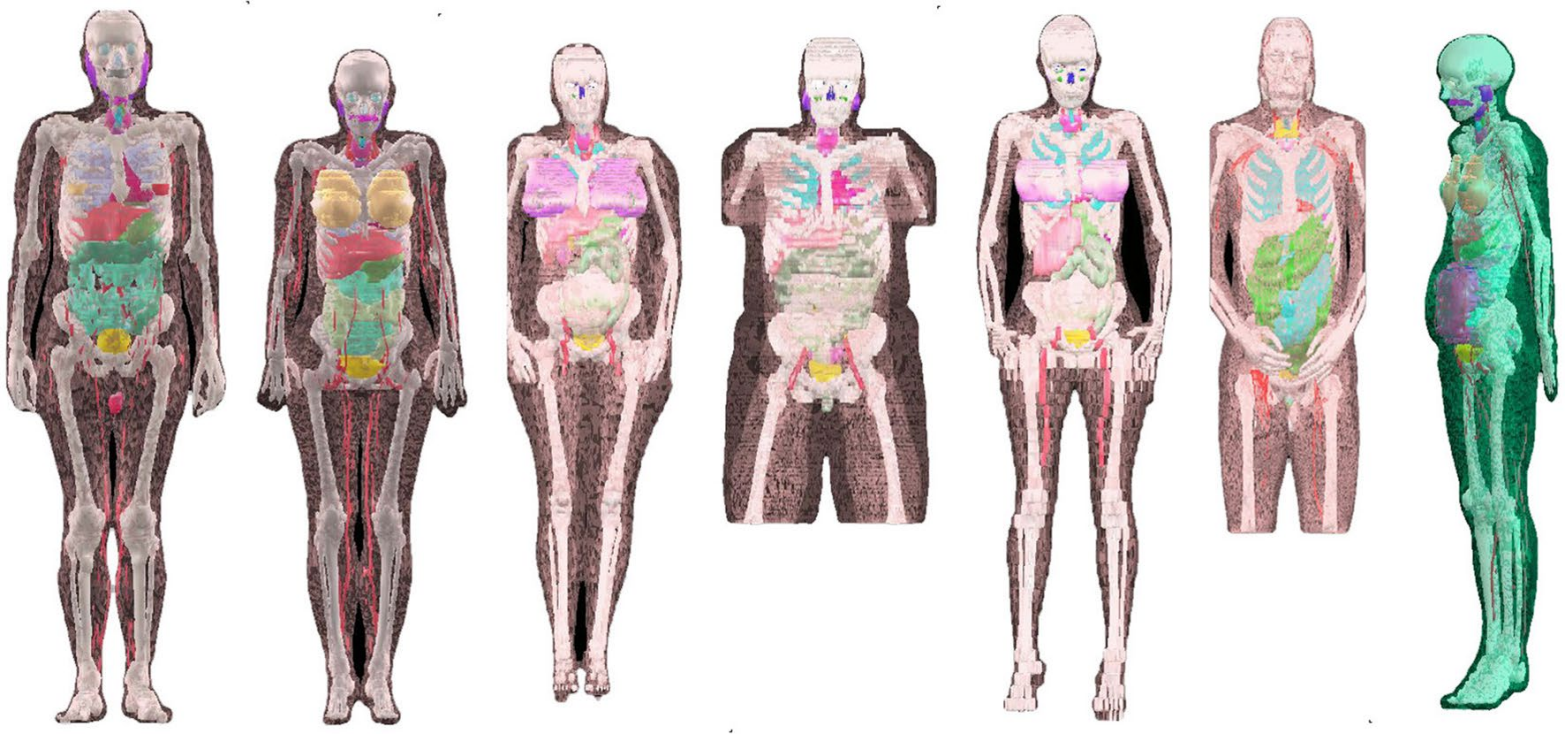
Voxel phantoms (from left to right): ICRP reference male, ICRP reference female, Donna, Helga, Irene, Visible Human, and Katja

In the present study five voxel phantoms representing individuals, one male and four females, were previously constructed at Helmholtz Zentrum München, Germany (Petoussi-Henss et al. 2002; Becker et al. 2008; Zankl 2010), were considered (Fig. 1). Furthermore, the ICRP reference male and female phantoms were also considered. Table 1 shows the characteristics of the used phantoms. The aim of the present work was to estimate the variability of organ and effective dose for different phantoms. Since the effective dose is per definition estimated using both reference male and female phantoms representing the reference person (ICRP 2007), for the present study a “detriment-weighted dose coefficient” was calculated (Eq. 3):

3 $$ e_{{{\text{DW}}}} \left( \tau \right) = \mathop \sum \limits_{T} w_{{\text{T}}} h\left( {r_{{\text{T}}} ,\tau } \right)^{{{\text{Male}}/{\text{Female}}}} . $$

**Table 1:** Phantom characteristics

Characteristic	ICRP reference male^a^	ICRP reference female^a^	Donna^b^	Helga^b^	Irene^b^	Visible human^b^	Katja^c^
Sex	M	F	F	F	F	M	F
Age (year)	38	43	40	26	32	38	43
Hight (cm)	176	167	176	170	163	180	163
Mass (kg)	73	60	79	81	51	103	62.3
No. of voxels (million)	1.9	3.9	2.2	8.3	3.0	20.1	4.0
Voxel width (mm)	2.137	1.775	1.875	0.98	1.875	0.91	1.775
Voxel length (mm)	2.137	1.775	1.875	0.98	1.875	0.94	1.775
Voxel height (mm)	8	4.84	10	10	5	5	4.84
Coverage	Whole body	Whole body	Whole body	Head to thigh; no arms	Whole body	Head to thigh	Whole body and Fetus 24 week of gestation

*M* male, *F* female

^a^ICRP (2009)

^b^Zankl (2010)

^c^Becker et al. (2008)

Due to the sampling method used in the present study, the detriment-weighted dose coefficient was calculated here by using $h\left( {r_{{\text{T}}} ,\tau } \right)^{{{\text{Male}}/{\text{Female}}}}$—the equivalent dose coefficients for a randomized mixture for male and female.

Nevertheless, the tissue-weighting factors $w_{{\text{T}}}$ for sex-specific organs were used.

### Computation of *S* values

The *S* values $S\left( {r_{{\text{T}}} \leftarrow r_{{\text{S}}} } \right)$, which express the dose to the target organ $r_{{\text{T}}}$, per unit accumulated activity of a particular radionuclide in source organ $r_{{\text{S}}}$, were calculated using an in house software package based on SAFs pre-calculated SAFs using various voxel phantoms. In contrast to other software packages that utilize a single adult male and a single adult female stylized phantom of reference size, the HMGU software employs the HMGU’s library of pre-calculated SAF values for photons and electrons, based on several anthropomorphic adult phantoms of reference and non-reference size (Virtual Human Database) as well as on the ICRP SAF values of Publication 133 (ICRP 2016). For any radionuclide, the software uses gamma-ray energies and detailed beta spectra as given in the electronic nuclear database accompanying ICRP Publication 107 (ICRP 2008).

The *S* value can be calculated according to the following formula:

4 $$ S_{{\text{w}}} \left( {r_{{\text{T}}} \leftarrow r_{{\text{S}}} } \right) = \mathop \sum \limits_{R} w_{R} \mathop \sum \limits_{i} E_{i} Y_{i} \frac{{\phi \left( {r_{{\text{T}}} \leftarrow r_{{\text{S}}} ,E_{i} } \right)}}{{M\left( {r_{{\text{T}}} } \right)}}, $$

where $w_{{\text{R}}}$ is the radiation-weighting factor, $E_{i}$ is the mean energy of radiation type $i$, $Y_{i}$ is the yield of radiation type $i$ per nuclear transformation, $\phi \left( {r_{{\text{T}}} \leftarrow r_{{\text{S}}} ,E_{i} } \right)$ is the so-called absorbed fraction (AF), the fraction of energy emitted in the source tissue $r_{{\text{S}}}$ that is absorbed in the target tissue $r_{{\text{T}}}$, and $M\left( {r_{{\text{T}}} } \right)$ is the mass of the target tissue. Specific absorbed fraction $\Phi \left( {r_{{\text{T}}} \leftarrow r_{{\text{S}}} ,E_{i} } \right)$ is defined as absorbed fraction per unit mass and is averaged over the entire volume of the target organ (acronym SAF).

Calculations of SAFs were performed using the Monte Carlo radiation transport code EGSnrc (Kawrakow et al. 2009), to follow photon or electron particle histories originating in a source tissue $r_{{\text{S}}}$. The interaction processes of particles in the phantoms considered in the Monte Carlo simulation were photoelectric absorption, Compton scattering and pair production. For all phantoms, SAFs for photons were calculated. For electrons, SAFs obtained with Monte Carlo methods were considered for the following phantoms: ICRP reference male, ICRP reference female, Visible Human, and Katja.

Photon transport was terminated when the photon energy was below 2 keV. The secondary electrons generated by the interaction between the primary photons and target tissues were transported until their kinetic energy was below 20 keV.

Since the tiny bone marrow cavities cannot be resolved by the voxel dimensions used in the voxel phantoms, an indirect method of bone dosimetry had to be applied. Bone voxels are assumed to consist of a mixture of red and yellow bone marrow and trabecular mineral bone. The amount of energy deposited in a bone voxel during a photon interaction event is then partitioned to the individual bone components according to their mass proportions and mass energy-absorption coefficients (for the photon energy before the interaction). For active (red) bone marrow and bone endosteum, additional correction factors are applied which account for the extra photo-electrons produced in the bone trabeculae that enter the marrow cavities. These correction factors differ between the various bone groups. Details of the method of bone marrow and endosteum dosimetry depended on the phantom. For the ICRP reference adult male and female phantom as well as for the Katja phantom, the relative red bone marrow content and marrow cellularity (i.e., the fraction of marrow that is still haematopoietically active) of different bone groups (ICRP 1995) were considered, and the dose enhancement factors from Johnson et al. (2011) were used. For the other phantoms (Donna, Helga, Irene and Visible Human), the marrow fraction of each bone voxel was estimated from the original CT number, an equal fraction of red and yellow marrow was assumed, and dose enhancement factors from King and Spiers (1985) were used.

For all other electron SAFs, the following approximations were used (ICRP 1979):

5 $$ \Phi \left( {r_{{\text{T}}} \leftarrow r_{{\text{S}}} } \right) = \left\{ {\begin{array}{*{20}l} {1/M_{{\text{T}}} \quad {\text{for}}\,r_{{\text{T}}} = r_{{\text{S}}} } \\ {0\quad {\text{for}}\,r_{{\text{T}}} \ne r_{{\text{S}}} } \\ {0.5/M_{{\text{c}}} \quad {\text{for}}\,r_{{\text{T}}} = {\text{wall}},\,r_{{\text{S}}} = {\text{content}}} \\ {1/M_{{{\text{TB}}}} \quad {\text{for}}\,r_{{\text{s}}} = {\text{Totalbody,}}} \\ \end{array} } \right. $$

where $M_{{\text{c}}}$ is the mass of the contents of a walled organ, ${\text{TB}}$ is the total body, and $M_{{\text{T}}}$ and $M_{{{\text{TB}}}}$ are the masses of the target region and the total body, respectively.

As shown in the Fig. 1 and Table 1 the coverage of the phantoms was not the same. Also, some organs of Donna, Helga, Irene and visible human like breast, salivary glands, endosteum, heart wall, lymphatic nodes and oral mucosa have not been segmented and were therefore represented by ‘surrogate’ organs that have approximately the same anatomical position and size (Table 2).

**Table 2:** Surrogate organs

Target organ	Phantom	Surrogate organ
Breast	Visible human Donna, Helga, Irene	Breast ICRP reference male
Salivary glands	Donna, Helga, Irene	Salivary glands ICRP reference female
Endosteum	Visible human Donna, Helga, Irene	Skeleton
Heart wall	Donna, Helga, Irene	Total heart
Lymphatic nodes	Visible human Donna, Helga, Irene	Lymphatic nodes ICRP reference male Lymphatic nodes ICRP reference female
Oral mucosa	Visible human Donna, Helga, Irene	Oral mucosa ICRP reference male Oral mucosa ICRP reference female

As expected, the different organ topology, organ shape, and size in the anthropomorphic phantoms cause differences in the calculated SAF values. But also differences between the dosimetric methods—electron approximation, full or partial body coverage, surrogate regions, differences on skeletal dosimetry (Zankl et al. 2012; ICRP 2016)—can result in large deviations of SAF values for some source-target pairs. The comparison of the uncertainties of the biokinetic and dosimetric parameters could lead to the impression that the contribution of the SAFs to the uncertainty of the dose coefficients is much higher than that of the biokinetic parameters. To exclude the contribution to the uncertainties from the differences in the dosimetric methods mentioned above, calculations with three phantoms (ICRP reference male and female (ICRP 2009) and Katja (Becker et al. 2008)) were also performed. For these phantoms, the same dosimetric methods were used in determination of the SAF values.

### Biokinetic model

The transport of activity in the human body is described by a radionuclide-specific biokinetic model. The compartments of the model, which are the source organs for the activity, are linked to each other by biokinetic parameters. The time-integrated activity in each compartment is calculated by solving a system of ordinary linear differential equations with biokinetic parameters $k$:

6 $$ \frac{{{\text{d}}q_{i} \left( t \right)}}{{{\text{d}}t}} = \dot{I}\left( t \right) - \mathop \sum \limits_{j = 0,j \ne i}^{n} k_{ji} q_{i} \left( t \right) - \lambda_{{\text{p}}} q_{i} \left( t \right) + \mathop \sum \limits_{j = 1,j \ne i}^{n} k_{ij} q_{j} \left( t \right), $$

where $q_{i} \left( t \right)\left[ {Bq} \right]$ is the activity of the radioactive substance in compartment $i$ at the time $t$; $\dot{I}\left( t \right)\left[ {Bq \cdot d^{ - 1} } \right]$ is the rate of input from outside of the system; $k_{ji} \left[ {d^{ - 1} } \right]$ is the transfer coefficient from compartment $i$ to $j$; $k_{ij} \left[ {d^{ - 1} } \right]$ is the transfer coefficient of substance transferred from $j$ to $i$; $k_{0i}$ is the coefficient of loss rate to outside of the system; and $\lambda_{{\text{p}}}$ is the radioactive decay constant (Berman 1976;1–14).

The solution of this system of differential equations provides the activity $q_{i} \left( t \right)$ in each compartment, and the time-integrated activity in a source organ $\tilde{A} = \int_{0}^{{T_{{\text{D}}} }} q \left( t \right){\text{d}}t$ can be calculated. The dose-integration period $T_{{\text{D}}}$ is typically taken to be 50 years.

**Fig. 2:**
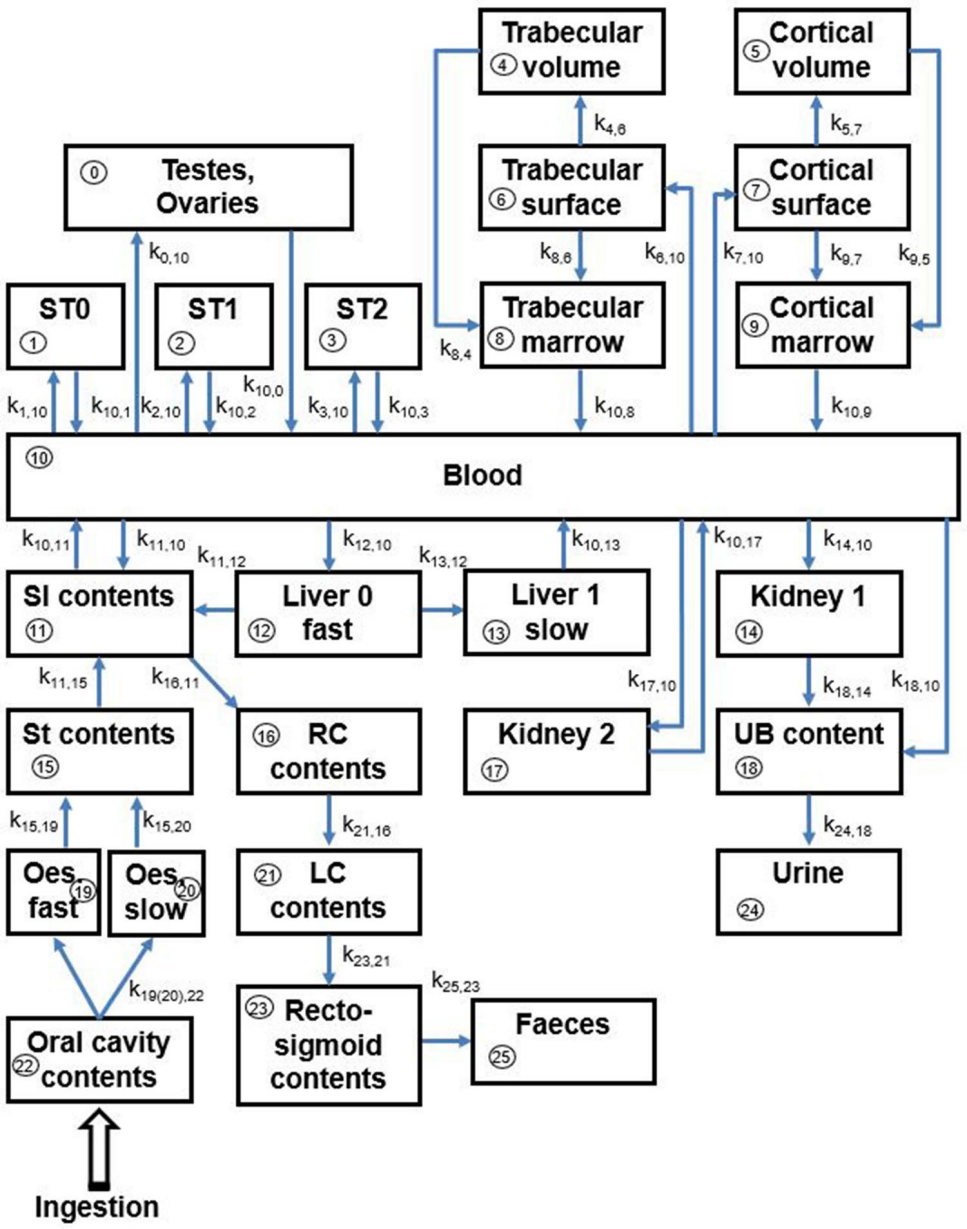
Compartment model for lanthanides used in the present study. *ST* soft tissue, *SI* small intestine, *St* stomach, *RC* right colon, *LC* left colon, *UB* urinary bladder, *Oes* oesophagus

The compartment model that describes the behavior of lanthanides in the human body and its corresponding model parameters $k$ have been reported by Taylor et al. (1998, 2003). Based on these publications the biokinetic model of ^141^Ce was created (Fig. 2). In the present study only ingestion of radionuclides was considered. For this reason, the model was extended to include the esophagus as a compartment. By solving the system of ordinary linear differential equations (Eq. 6) the time-integrated activity $\tilde{A}$ in each compartment was calculated.

Because of many unstable daughter nuclides, the biokinetic model of ^144^Ce is more complicated than that of ^141^Ce. For the daughter nuclides ^144m^Pr and ^144^Pr the biokinetic models have also to be defined and linked by the physical decay constant $\lambda_{{\text{p}}}$. Because the biokinetic behavior of the light lanthanides Lanthanum, Cerium and Praseodymium is sufficiently similar (Taylor and Leggett 1998, 2003), they can be described by the same compartment model and parameter values. Because of the very long half-life of ^144^Nd (*t*_1/2_ = 2.29 × 10^15^ year) in the decay chain of ^144^Ce, the ^144^Nd was considered as stable.

### Determination of uncertainty of biokinetic parameters

The determination of the uncertainties of the dose coefficients was carried out numerically. It was assumed that biokinetic and dosimetric parameters are statistical values with the normal or lognormal distribution. Solutions of models with parameters sampled from the according distributions have been inserted in Eq. 1.

For a sampling of the biokinetic parameters, the Latin hypercube sampling method (LHS) (Iman and Shortencarier 1984) was used. To generate the samples this technique requires minimum and maximum values and the type of distribution of the parameters. For $n$ samples, LHS divides the range between the minimum and maximum values of each parameter into $n$ intervals on the basis of equal probability. With respect to the probability density in the interval, one value from each interval will be selected randomly. The samples thus obtained for the first parameter were paired in a random manner with the samples of the second parameter, then with the third parameter and so forth until $n$ $m$-tuples can be formed. This results in a $n \times m$ matrix of input in which the $i$th row contains values of each of the $m$ input variables to be used on the $i$th run ($n$ runs) of the computer model.

The biokinetic parameters (i.e. transfer coefficients) $k_{ij}$ (per day) can be related to the half-time $T_{j}$ of removal from the compartment $j$ according to the following formula:

7 $$ k_{ij} = \frac{\ln 2}{{T_{j} }}F_{ij} , $$

where $F_{ij}$ is the deposition fraction of activity in compartment $i$ transferred from compartment $j$. The variables $k_{ij}$, $T_{j}$ and $F_{ij}$ can be found in various publications (Taylor and Leggett 1998, 2003; ICRP 2006).

The distribution of the biokinetic parameters is unknown. The assumption that they are, as other physiological parameters, log-normally distributed, is not verifiable. Studies on the influence of the distributions of the biokinetic parameters on calculated radiation doses have shown that models whose parameters follow different distribution functions do not provide substantially different results (Klein 2011).

In the present study, it was assumed that these variables are normally distributed statistical values. For the biokinetic parameters for which there was insufficient information on how to base an estimate of the uncertainty, a coefficient of variation $c_{{\text{v}}}$ of 20% was assumed. For a confidence interval of 95%, the coefficient of variance of 20% corresponds to a coverage probability of more than 99.2% (Sappakitkamjorn and Niwitpong 2013). The standard deviation $\sigma$ was calculated for all statistical values according to Eq. 8:

8 $$ c_{{\text{v}}} = \frac{\sigma }{\mu }, $$

where $\mu$ is the mean value.

By calculating $\sigma$ values for the variables $T_{i}$, $F_{ij}$ and ${\text{DF}}_{ij}$ and with the propagation of uncertainty, the standard deviation for $k$ was further estimated.

Based on a normal distribution and a confidence interval of 95%, the minimum and maximum values (97.5th and 2.5th percentiles of the normal distribution) of the model parameters $k$ for the Latin hypercube sampling were estimated to be (Eqs. 9 and 10):

9 $$ {\text{Minimum}} = \mu - 1.96\sigma , $$

10 $$ {\text{Maximum}} = \mu + 1.96\sigma . $$

### Determination of uncertainty of *S* values

As described above, tables of *S* values were created for ^141^Ce, ^144^Ce, ^144m^Pr and ^144^Pr, and the seven voxel phantoms used in the present study. For each source-target pair of organs, up to seven *S* values were generated (a couple of organs were not available in all phantoms, therefore not for every organ pair *S* values could be evaluated).

First it was assumed that these *S* values are normally distributed. The corresponding mean value and standard deviation for this distribution were calculated. Based on the confidence interval of 95%, the minimum and maximum values were determined according to Eqs. 9 and 10.

For those organ pairs for which this method would introduce negative *S* values, a log-normal *S* value distribution was assumed. The geometric mean value $\mu^{*}$ and the geometric standard deviation $\sigma^{*}$ were determined and the minimum and maximum values (97.5th and 2.5th percentiles of the log-normal distribution) were recalculated according to Eqs. 11 and 12:

11 $$ {\text{Minimum}} = \mu^{*} \div \left( {\sigma^{*} } \right)^{1.96} , $$

12 $$ {\text{Maximum}} = \mu^{*} \times \left( {\sigma^{*} } \right)^{1.96} . $$

### Determination of uncertainty of dose coefficients

Based on the Rosenbrock method (Rosenbrock 1963) for solving a system of stiff ordinary differential equations, a computer code called UnDose, written in C#, was developed for the present work, for calculating the uncertainty of the dose coefficients for ^141^Ce and ^144^Ce according to Eqs. 1 and 3.

Five hundred sample values of the biokinetic and dosimetric parameters generated with the LHS method were used as input for the software. As output, 500 samples of activity $q_{i} \left( t \right)$ (Eq. 6) and time-integrated activities $\tilde{A}$ (Eq. 1) in each compartment and 500 samples of distribution of equivalent (Eq. 1) and detriment weighted dose (Eq. 3) coefficients were calculated. These values reflect the uncertainty of the dose coefficients and can be used for calculating any statistical values—mean values, standard deviations, percentiles, etc.

To quantify the uncertainty of dose coefficients the uncertainty factor UF (Leggett 2001; Li et al. 2011, 2015; Puncher 2014) was used. The uncertainty-associated quantity can be expressed in terms of lower and upper bounds, A and B, respectively. The UF for a confidence interval of 95% is defined as the square root of the ratio between 97.5th (B) and 2.5th (A) percentiles.

## Results

**Fig. 3:**
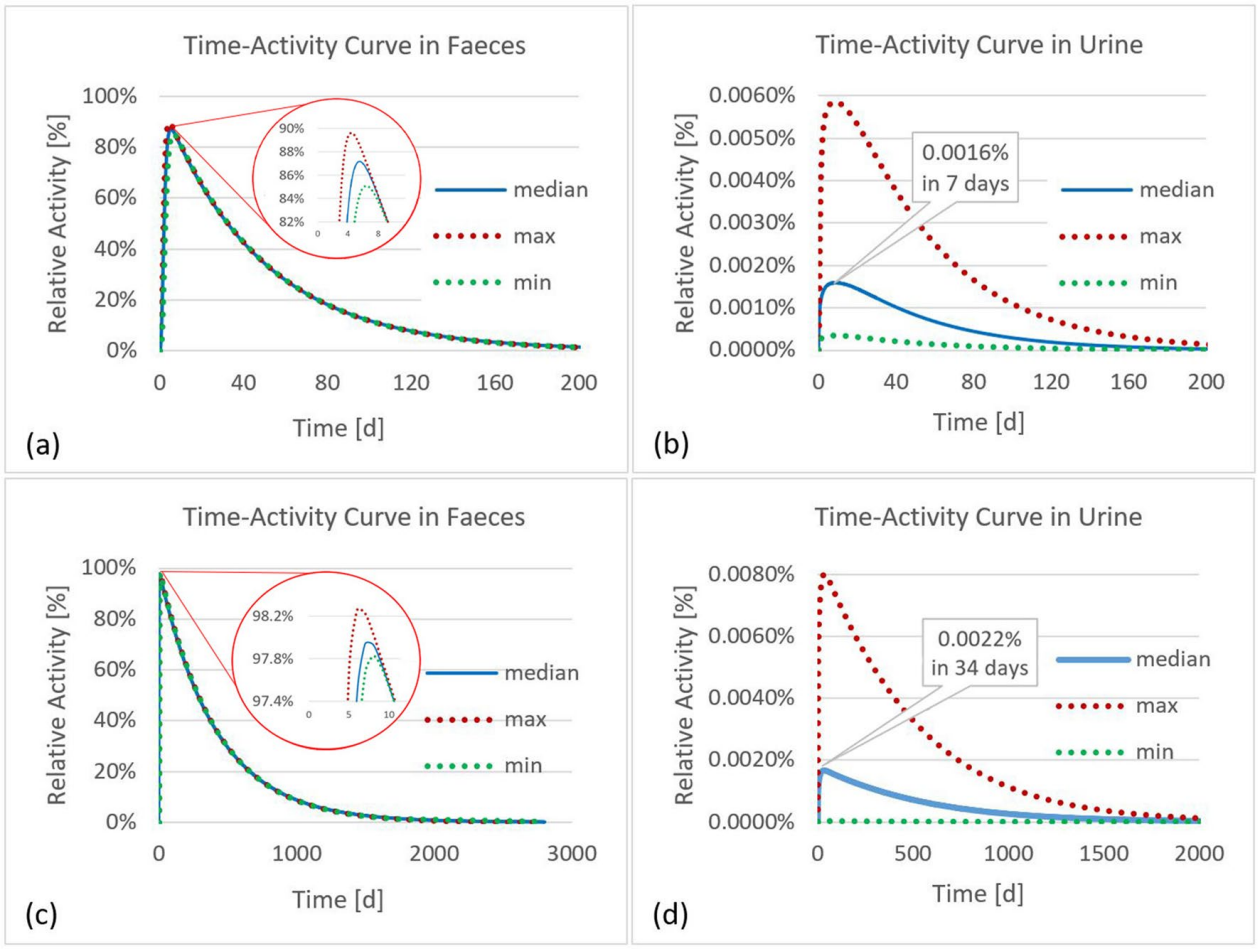
Time-activity curves in feces and urine for the biokinetic model of ^141^Ce (**a**, **b**) and ^144^Ce (**c**, **d**)

**Fig. 4:**
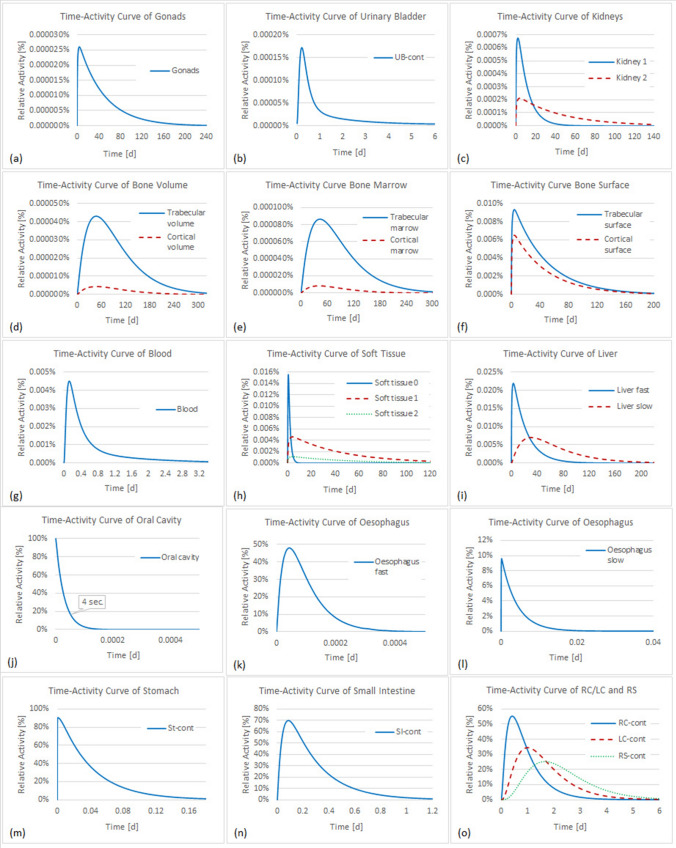
Time-activity curves in compartments of the biokinetic model of ^141^Ce. *UB* urinary bladder, *St-cont* stomach contents, *SI-cont* small intestine contents, *RC-cont* right colon contents, *LC-cont* left colon contents, *RS-cont* rectosigmoid contents

**Fig. 5:**
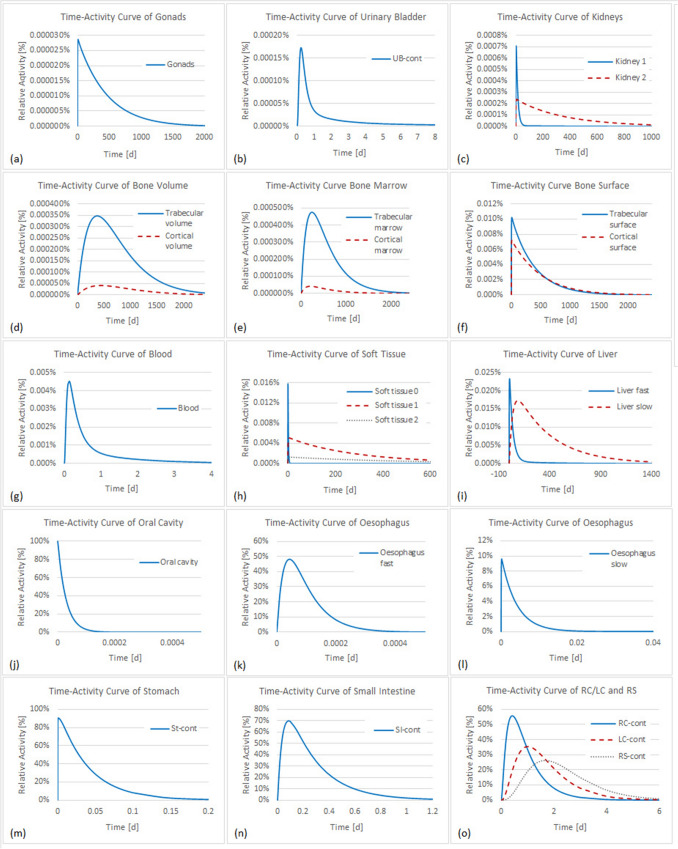
Time-activity curves in compartments of the biokinetic model of ^144^Ce. *UB* urinary bladder, *St-cont* stomach contents, *SI-cont* small intestine contents, *RC-cont* right colon contents, *LC-cont* left colon contents, *RS-cont* rectosigmoid contents

Figures 3, 4 and 5 show the time-dependent activity in urine, feces, and all other compartments of the biokinetic model for ^141^Ce and ^144^Ce. As mentioned above 500 samples of activity in each compartment were calculated. However, for a better overview the activities in Figs. 4 and 5 are shown for only one sample. For better monitoring the activities in Fig. 3 are shown for 97.5th and 2.5th percentiles and for median values.

**Fig. 6:**
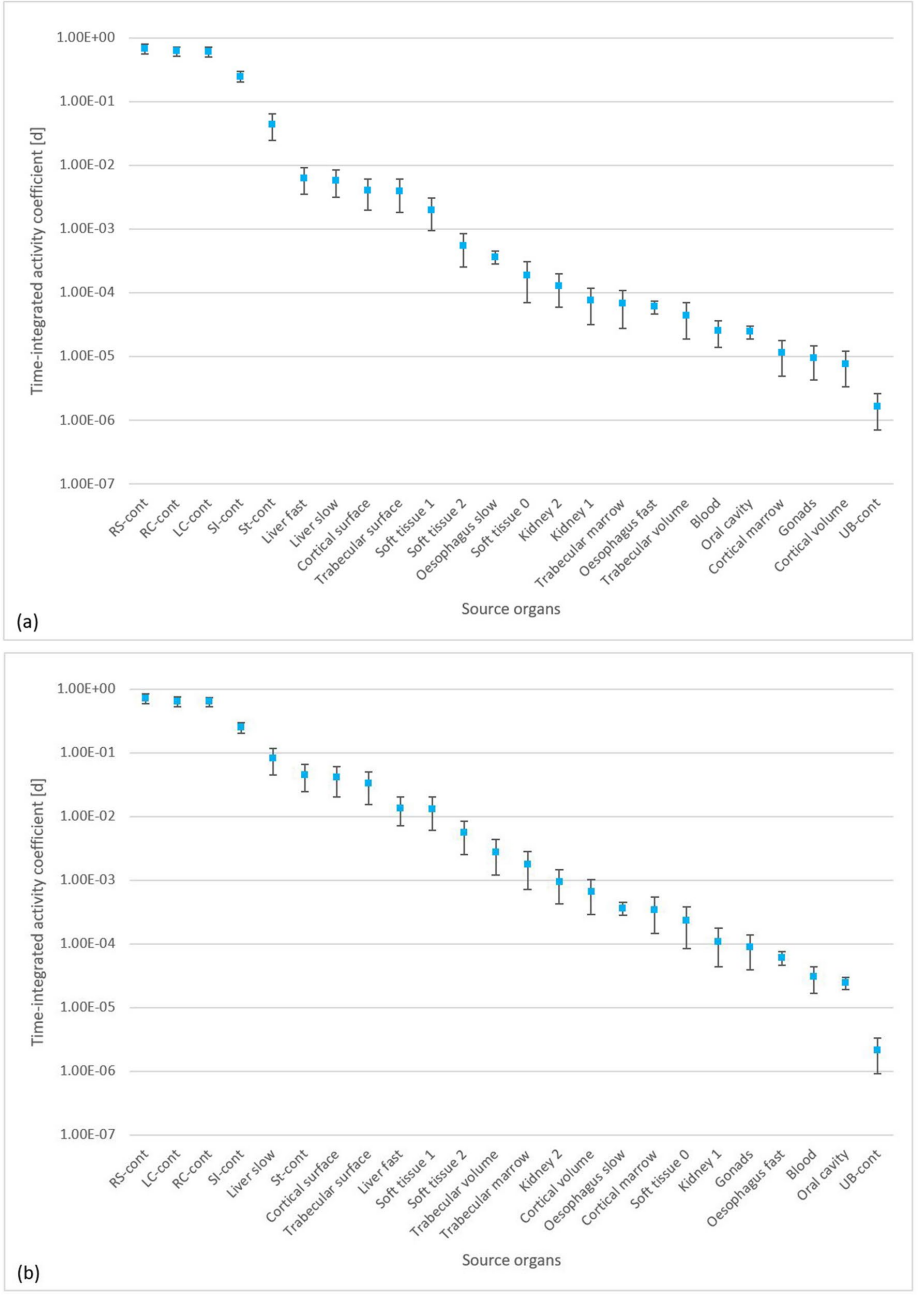
Time-integrated activity coefficients for the compartments of the biokinetic model of ^141^Ce (**a**) and ^144^Ce (**b**). *RC-cont* right colon contents, *LC-cont* left colon contents, *RS-cont* rectosigmoid contents, *SI-cont* small intestine contents, *St-cont* stomach contents, *UB* urinary

Figure 6 shows the source organs with their corresponding time-integrated activities per unit ingested activity for ^141^Ce and ^144^Ce. This normalized time-integrated activity has units of time and therefore is often referred to as "residence time". As previously reported (ICRP 1959), for the ingestion of ^141^Ce and ^144^Ce the most critical organ is the colon and, accordingly, the present results demonstrate that the cumulative activity for the colon is the highest.

The calculated minimum and maximum values of the statistically distributed biokinetic parameters for Cerium are summarized in Table 3. The minimum and maximum values are the 2.5th and 97.5th percentiles of the distribution of each parameter, respectively. It was assumed that the biokinetic parameters were normally distributed. The coefficient of variation varies here from 15.91 to 31.64%.

**Table 3:** Uncertainty of the biokinetic parameters of Ce in (day^−1^)

	Min	Mean	Max		Min	Mean	Max
*k*_12,10_	8.5 × 10^–1^	1.2 × 10^1^	2.2 × 10^1^	*k*_5,7_	1.6 × 10^–5^	4.0 × 10^–5^	6.7 × 10^–5^
*k*_1,10_	5.2 × 10^–1^	1.0 × 10^1^	2.0 × 10^1^	*k*_9,5_	3.1 × 10^–5^	8.0 × 10^–5^	1.3 × 10^–4^
*k*_2,10_	1.4 × 10^–1^	1.9 × 10^0^	3.6 × 10^0^	*k*_10,8_	2.9 × 10^–3^	7.6 × 10^–3^	1.2 × 10^–2^
*k*_3,10_	3.4 × 10^–2^	4.7 × 10^–1^	9.0 × 10^–1^	*k*_8,6_	1.9 × 10^–4^	4.9 × 10^–4^	8.0 × 10^–4^
*k*_7,10_	2.6 × 10^–1^	3.5 × 10^0^	6.7 × 10^0^	*k*_4,6_	9.4 × 10^–5^	2.5 × 10^–4^	4.0 × × 10^–4^
*k*_6,10_	2.6 × 10^–1^	3.5 × 10^0^	6.7 × 10^0^	*k*_8,4_	1.9 × 10^–4^	4.9 × 10^–4^	8.0 × 10^–4^
*k*_14,10_	2.6 × 10^–2^	3.5 × 10^–1^	6.7 × × 10^–1^	*k*_18,14_	3.8 × 10^–2^	9.9 × 10^–2^	1.6 × 10^–1^
*k*_11,10_	1.0 × 10^–1^	1.4 × 10^0^	2.7 × 10^0^	*k*_10,17_	5.3 × 10^–4^	1.4 × 10^–3^	2.2 × 10^–3^
*k*_17,10_	8.5 × 10^–3^	1.2 × 10^–1^	2.2 × 10^–1^	*k*_10,0_	1.5 × 10^–4^	3.8 × 10^–4^	6.2 × 10^–4^
*k*_0,10_	5.9 × 10^–4^	8.2 × 10^–3^	1.6 × 10^–2^	*k*_24,18_	4.6 × 10^0^	12 × 10^0^	1.9 × 10^1^
*k*_18,10_	3.4 × 10^–2^	4.7 × 10^–1^	9.0 × 10^–1^	*k*_25,23_	7.1 × 10^–1^	1.5 × 10^0^	2.2 × 10^0^
*k*_10,13_	3.6 × 10^–4^	9.5 × 10^–4^	1.5 × 10^–3^	*k*_23,21_	8.3 × 10^–1^	1.6 × 10^0^	2.4 × 10^0^
*k*_11,12_	7.1 × 10^–4^	2.3 × 10^–3^	3.9 × 10^–3^	*k*_21,16_	8.3 × 10^–1^	1.6 × 10^0^	2.4 × 10^0^
*k*_13,12_	7.9 × 10^–3^	2.1 × 10^–2^	3.4 × 10^–2^	*k*_16,11_	1.9 × 10^0^	4.1 × 10^0^	6.4 × 10^0^
*k*_10,1_	5.3 × 10^–1^	1.4 × 10^0^	2.24 × 10^0^	*k*_10,11_	5.1 × 10^–5^	2.3 × 10^–3^	4.5 × 10^–3^
*k*_10,2_	7.2 × 10^–4^	1.9 × 10^–3^	3.07 × 10^–3^	*k*_11,15_	3.5 × 10^0^	2.5 × 10^1^	4.7 × 10^1^
*k*_10,3_	4.8 × 10^–5^	1.3 × 10^–4^	2.05 × 10^–4^	*k*_15,19_	5.9 × 10^3^	1.6 × 10^4^	2.5 × 10^4^
*k*_10,9_	2.9 × 10^–3^	7.6 × 10^–3^	1.23 × 10^–2^	*k*_15,20_	1.1 × 10^2^	2.9 × 10^2^	4.7 × 10^2^
*k*_9,7_	3.1 × 10^–5^	8.0 × 10^–5^	1.33 × 10^–4^	*k*_19/20,22_	1.7 × 10^4^	4.3 × 10^4^	7.0 × 10^4^

Mean values of the first 29 parameters (*k*_12,10_*–k*_24,18_) are from Taylor and Leggett (2003)

Tables 4 and 5 show the calculated uncertainty of the *S* values of ^141^Ce, ^144^Ce and the daughter isotopes ^144^Pr and ^144m^Pr from seven phantoms, and from the three phantoms only (ICRP reference male, ICRP reference female, and Katja), respectively. The target organs from Table 6 and the source organs from Fig. 2 form the pairs of organs for which *S* values were calculated. The calculated minimum and maximum values and the type of distribution for the *S* values for every organ pair are not listed here explicitly for simplicity. Table 4 shows the range of uncertainty of normally and log-normally distributed *S* values. For a normal distribution, the coefficient of variation varies from 0.16 to 32.26%. For log-normally distributed *S* values, the geometric standard deviation was estimated and varies from 1.03 to 14.03.

**Table 4:** Uncertainty of the *S* values of ^141^Ce, ^144^Ce, ^144^Pr and ^144m^Pr calculated for the 7 phantoms of Table 1

	CV %			GSD		
	Distribution	Min (%)	Max (%)	Distribution	Min	Max
^141^Ce	Normal	0.29	32.16	Lognormal	1.03	9.11
^144^Ce	Normal	0.28	32.26	Lognormal	1.05	7.31
^144^Pr	Normal	0.16	32.03	Lognormal	1.28	14.03
^144m^Pr	Normal	0.39	32.17	Lognormal	1.27	8.81

*CV* coefficient of variation, *GSD* geometric standard deviation

**Table 5:** Uncertainty of the *S* values of ^141^Ce, ^144^Ce, ^144^Pr and ^144m^Pr calculated for the 3 phantoms (ICRP reference male, ICRP reference female, and Katja)

	CV %			GSD		
	Distribution	Min (%)	Max (%)	Distribution	Min	Max
^141^Ce	Normal	0.29	32.18	Lognormal	1.27	4.15
^144^Ce	Normal	0.62	31.35	Lognormal	1.28	7.31
^144^Pr	Normal	0.50	32.03	Lognormal	1.30	12.0
^144m^Pr	Normal	0.42	32.17	Lognormal	1.27	5.75

*CV* coefficient of variation, *GSD* geometric standard deviation

**Table 6:** Uncertainty of dose coefficients for ^141^Ce and ^144^Ce from the calculations with all seven phantoms

Target	^141^Ce in (Sv/Bq)				^144^Ce in (Sv/Bq)			
	Mean	SD	UF	ICRP*	Mean	SD	UF	ICRP*
Brain	2.77E−13	1.01E−13	2.00	2.20E−13	6.93E−13	3.11E−13	2.47	1.45E−11
Breast	3.34E−12	6.74E−13	1.46	2.90E−12	1.17E−12	3.26E−13	1.69	8.10E−12
Colon	1.85E−09	1.21E−09	3.08	2.40E−10	5.42E−10	5.42E−10	4.62	5.75E−09
Endosteum	1.61E−11	3.88E−12	1.53	1.40E−11	4.60E−11	2.16E−11	2.38	1.65E−10
Liver	4.81E−11	1.07E−11	1.52	3.05E−11	8.49E−11	3.77E−11	2.34	7.25E−10
Lungs	5.12E−12	9.45E−13	1.43	3.55E−12	1.69E−12	3.97E−13	1.57	1.65E−11
Oesophagus	4.80E−11	1.28E−11	1.65	1.15E−11	1.97E−11	4.49E−12	1.50	2.40E−11
Red marrow	1.99E−11	2.53E−12	1.27	1.75E−11	1.52E−11	5.22E−12	1.83	1.25E−10
Salivary glands	3.51E−13	9.16E−14	1.64	3.15E−13	6.05E−13	2.63E−13	2.41	1.17E−11
Skin	4.66E−12	4.69E−13	1.20	3.70E−12	1.72E−12	2.86E−13	1.37	7.35E−12
Stomach wall	3.99E−10	6.34E−10	5.06	1.65E−10	1.73E−10	3.62E−10	7.38	1.60E−09
Testes	1.18E−12	4.48E−13	2.00	9.50E−13	4.34E−12	2.14E−12	2.64	1.55E−11
Thyroid	1.02E−12	1.87E−13	1.44	6.75E−13	7.21E−13	2.65E−13	2.06	6.05E−12
Urinary bladder wall	2.10E−11	8.49E−12	1.96	4.40E−11	2.13E−11	4.66E−12	1.51	2.61E−10
Adrenals	3.56E−11	6.58E−12	1.41	1.75E−11	8.25E−12	1.68E−12	1.49	7.55E−11
Extrathoracic airways	4.40E−13	1.02E−13	1.56	3.15E−13	6.67E−13	2.82E−13	2.34	1.30E−11
Gall bladder wall	6.89E−11	2.25E−11	1.83	4.00E−11	1.44E−11	4.92E−12	1.88	4.30E−10
Heart wall	8.03E−12	1.55E−12	1.43	6.70E−12	2.51E−12	5.69E−13	1.56	2.50E−11
Kidneys	5.71E−11	8.24E−12	1.30	3.20E−11	1.57E−11	3.37E−12	1.50	8.20E−11
Lymph	4.74E−11	4.39E−12	1.20	3.20E−11	1.55E−11	1.47E−12	1.20	3.55E−10
Muscle	1.31E−11	1.61E−12	1.26	8.90E−12	4.41E−12	5.60E−13	1.26	2.35E−11
Oral mucosa	9.46E−13	5.33E−13	2.24	9.85E−13	9.67E−13	4.57E−13	2.38	2.15E−11
Pancreas	7.71E−11	1.42E−11	1.42	5.15E−11	1.61E−11	3.81E−12	1.55	2.05E−10
Prostate	2.71E−12	3.06E−13	1.24	6.50E−12	5.43E−12	1.03E−12	1.42	7.00E−12
Small intestine wall	3.45E−10	2.33E−10	2.92	1.35E−10	1.32E−10	1.23E−10	3.81	1.60E−09
Spleen	2.52E−11	7.34E−12	1.75	1.80E−11	6.06E−12	1.92E−12	1.78	1.90E−11
Thymus	2.50E−12	5.62E−13	1.56	1.19E−12	1.05E−12	3.23E−13	1.87	1.25E−11
Effective dose	2.85E−10	1.65E−10	2.54	6.20E−11	1.09E−10	1.13E−10	3.90	9.80E−10

The values of ICRP (2019) are also shown

^a^Averaged values over male and female (ICRP 2019)

*SD* standard deviation, *UF* uncertainty factor (unitless)

As shown in Table 5 for normal distribution the coefficient of variation varies from 0.29 to 32.18%. For log-normally distributed *S* values, the geometric standard deviation ranges from 1.27 to 7.31.

The uncertainty factors UF of the dose coefficients and the reference values from ICRP (ICRP 2019) of ^141^Ce and ^144^Ce are presented in Table 6.

For the isotope ^141^Ce, the uncertainty of the equivalent dose coefficients in the target organs ranges from 1.20 to 5.06, and the UF for the detriment-weighted dose coefficient is 2.54. For ^144^Ce, the uncertainty of the equivalent dose coefficients ranges from 1.20 to 7.38, and the UF for the detriment weighted dose coefficient is 3.90.

**Fig. 7:**
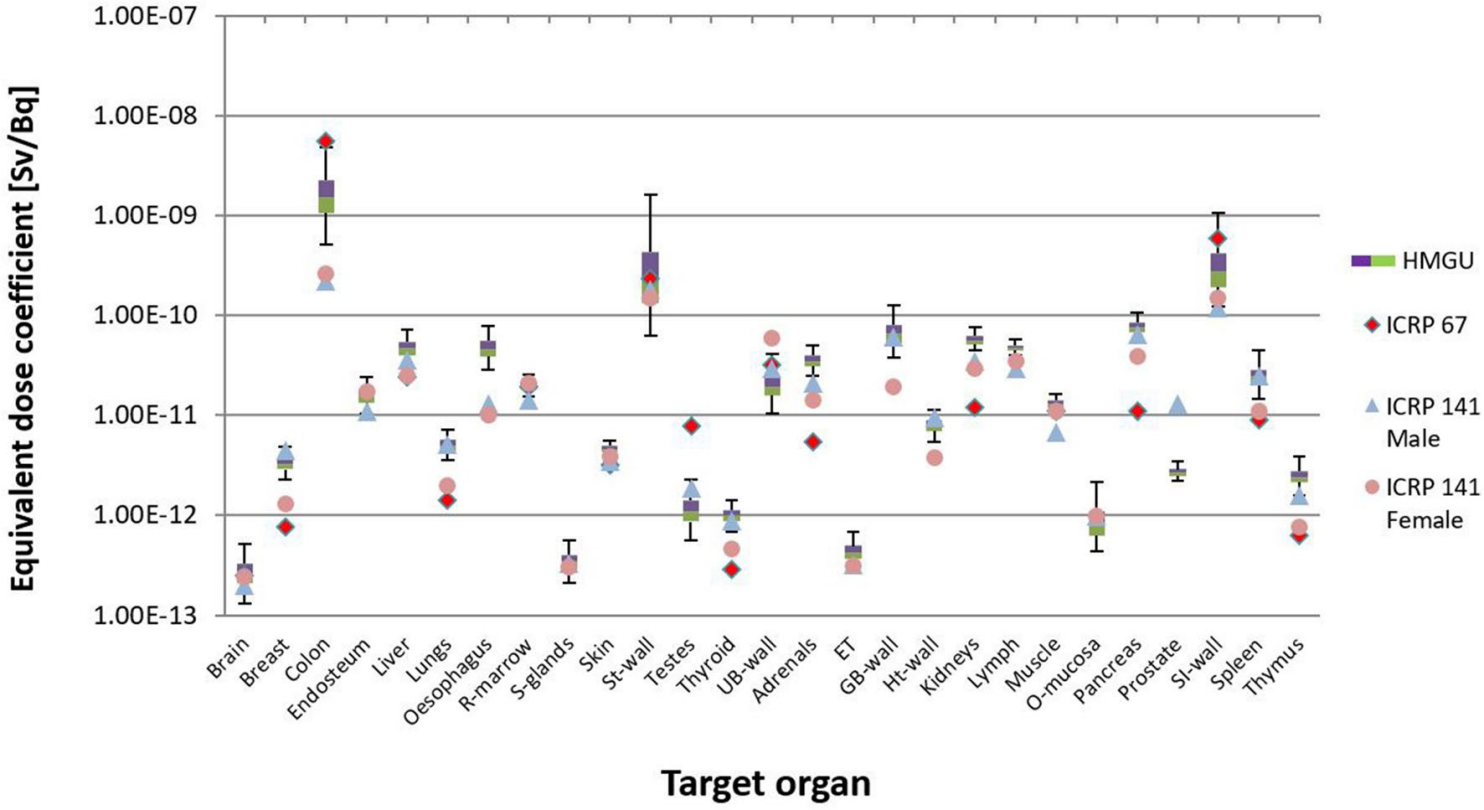
Calculated organ equivalent dose coefficients and the respective uncertainties for ^141^Ce from the calculation with all seven phantoms of Table 1. The values of ICRP (1993, 2019) are also shown

**Fig. 8:**
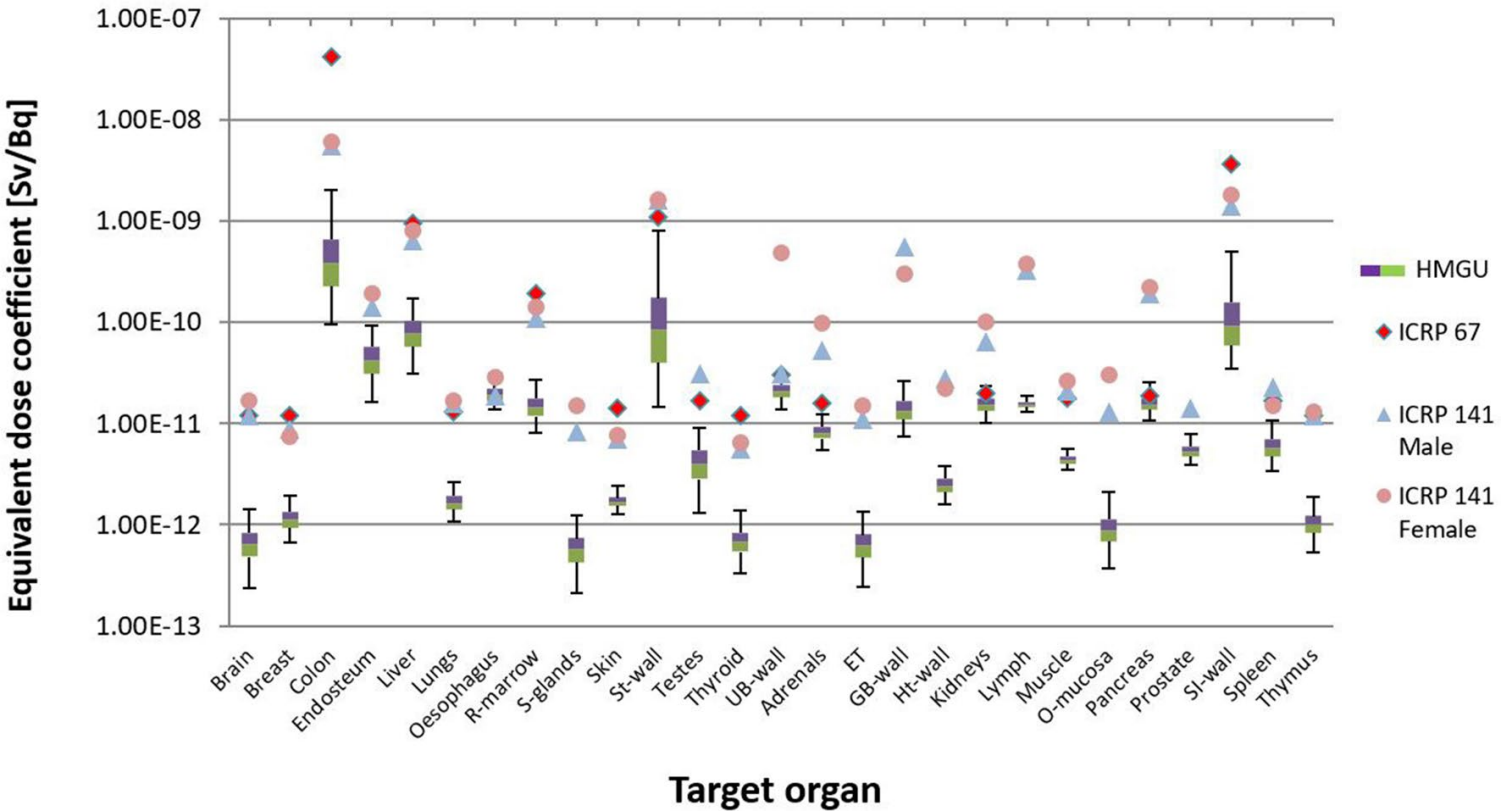
Uncertainty of dose coefficients for ^144^Ce from the calculation with all seven phantoms of Table 1. The values of ICRP (1993, 2019) are also shown

To compare the calculated values with the reference values from ICRP (ICRP 1993, 2019), the uncertainties of dose coefficients for ^141^Ce and ^144^Ce are shown in Figs. 7 and 8 in the form of boxplots. The whole range from the upper and lower ends of the whiskers reflects the 95% confidence interval, while within the box are the middle 50% of all values. The boundary line between the two colors of the box represents the median value.

The uncertainties of the dose coefficients of ^141^Ce and ^144^Ce for the three phantoms mentioned above are presented in Table 7. For ^141^Ce, the uncertainty of the equivalent dose coefficients varies from 1.18 to 2.27 and the UF for the detriment weighted dose coefficient is 1.26. For ^144^Ce, the uncertainty of the equivalent dose coefficients ranges from 1.21 to 2.55 and the UF for the detriment weighted dose coefficient is 1.31.

**Table 7:** Uncertainty of dose coefficients for ^141^Ce and ^144^Ce from the calculations with three phantoms (ICRP reference male, ICRP reference female and Katja)

Target	^141^Ce in [Sv/Bq]				^144^Ce in [Sv/Bq]			
	Mean	SD	UF	ICRP^a^	Mean	SD	UF	ICRP^a^
Brain	2.57E−13	1.01E−13	2.13	2.20E−13	6.92E−13	3.11E−13	2.48	1.45E−11
Breast	2.70E−12	7.62E−13	1.69	2.90E−12	1.26E−12	3.54E−13	1.71	8.10E−12
Colon	3.50E−10	3.95E−11	1.22	2.40E−10	8.20E−11	1.16E−11	1.31	5.75E−09
Endosteum	1.84E−11	3.98E−12	1.46	1.40E−11	4.59E−11	2.16E−11	2.39	1.65E−10
Liver	4.08E−11	1.05E−11	1.62	3.05E−11	8.81E−11	3.82E−11	2.32	7.25E−10
Lungs	3.88E−12	9.18E−13	1.55	3.55E−12	1.72E−12	4.21E−13	1.62	1.65E−11
Oesophagus	5.12E−11	1.57E−11	1.79	1.15E−11	1.85E−11	4.10E−12	1.53	2.40E−11
Red marrow	2.34E−11	2.93E−12	1.27	1.75E−11	1.55E−11	5.25E−12	1.82	1.25E−10
Salivary glands	3.40E−13	9.30E−14	1.66	3.15E−13	6.11E−13	2.63E−13	2.39	1.17E−11
Skin	4.75E−12	4.36E−13	1.18	3.70E−12	1.75E−12	2.84E−13	1.36	7.35E−12
Stomach wall	1.17E−10	6.65E−11	2.27	1.65E−10	3.38E−11	1.85E−11	2.21	1.60E−09
Testes	2.61E−12	5.22E−13	1.47	9.50E−13	3.73E−12	1.79E−12	2.55	1.55E−11
Thyroid	7.93E−13	1.53E−13	1.45	6.75E−13	7.18E−13	2.64E−13	2.04	6.05E−12
Urinary bladder wall	7.02E−11	1.46E−11	1.50	4.40E−11	2.10E−11	4.34E−12	1.50	2.61E−10
Adrenals	2.17E−11	3.84E−12	1.38	1.75E−11	7.67E−12	1.50E−12	1.47	7.55E−11
Extrathoracic airways	3.96E−13	1.00E−13	1.64	3.15E−13	6.68E−13	2.82E−13	2.35	1.30E−11
Gall bladder wall	4.27E−11	1.50E−11	1.85	4.00E−11	1.56E−11	4.95E−12	1.79	4.30E−10
Heart wall	6.51E−12	1.55E−12	1.53	6.70E−12	2.65E−12	6.29E−13	1.59	2.50E−11
Kidneys	4.62E−11	7.51E−12	1.37	3.20E−11	1.80E−11	3.62E−12	1.47	8.20E−11
Lymph	4.98E−11	4.77E−12	1.20	3.20E−11	1.52E−11	1.51E−12	1.21	3.55E−10
Muscle	1.22E−11	1.49E−12	1.25	8.90E−12	3.99E−12	5.24E−13	1.27	2.35E−11
Oral mucosa	9.26E−13	5.33E−13	2.26	9.85E−13	9.73E−13	4.56E−13	2.37	2.15E−11
Pancreas	6.54E−11	1.35E−11	1.46	5.15E−11	1.98E−11	4.11E−12	1.47	2.05E−10
Prostate	1.82E−11	3.58E−12	1.45	6.50E−12	5.45E−12	1.03E−12	1.42	7.00E−12
Small intestine wall	1.70E−10	3.10E−11	1.40	1.35E−10	4.54E−11	6.75E−12	1.32	1.60E−09
Spleen	1.92E−11	6.35E−12	1.72	1.80E−11	5.79E−12	1.92E−12	1.73	1.90E−11
Thymus	1.32E−12	2.68E−13	1.46	1.19E−12	9.10E−13	2.96E−13	1.93	1.25E−11
Effective dose	7.08E−11	9.88E−12	1.26	6.20E−11	2.38E−11	3.43E−12	1.31	9.80E−10

The values of ICRP (2019) are also shown

^a^Averaged values over male and female (ICRP 2019)

*SD* standard deviation, *UF* uncertainty factor

**Fig. 9:**
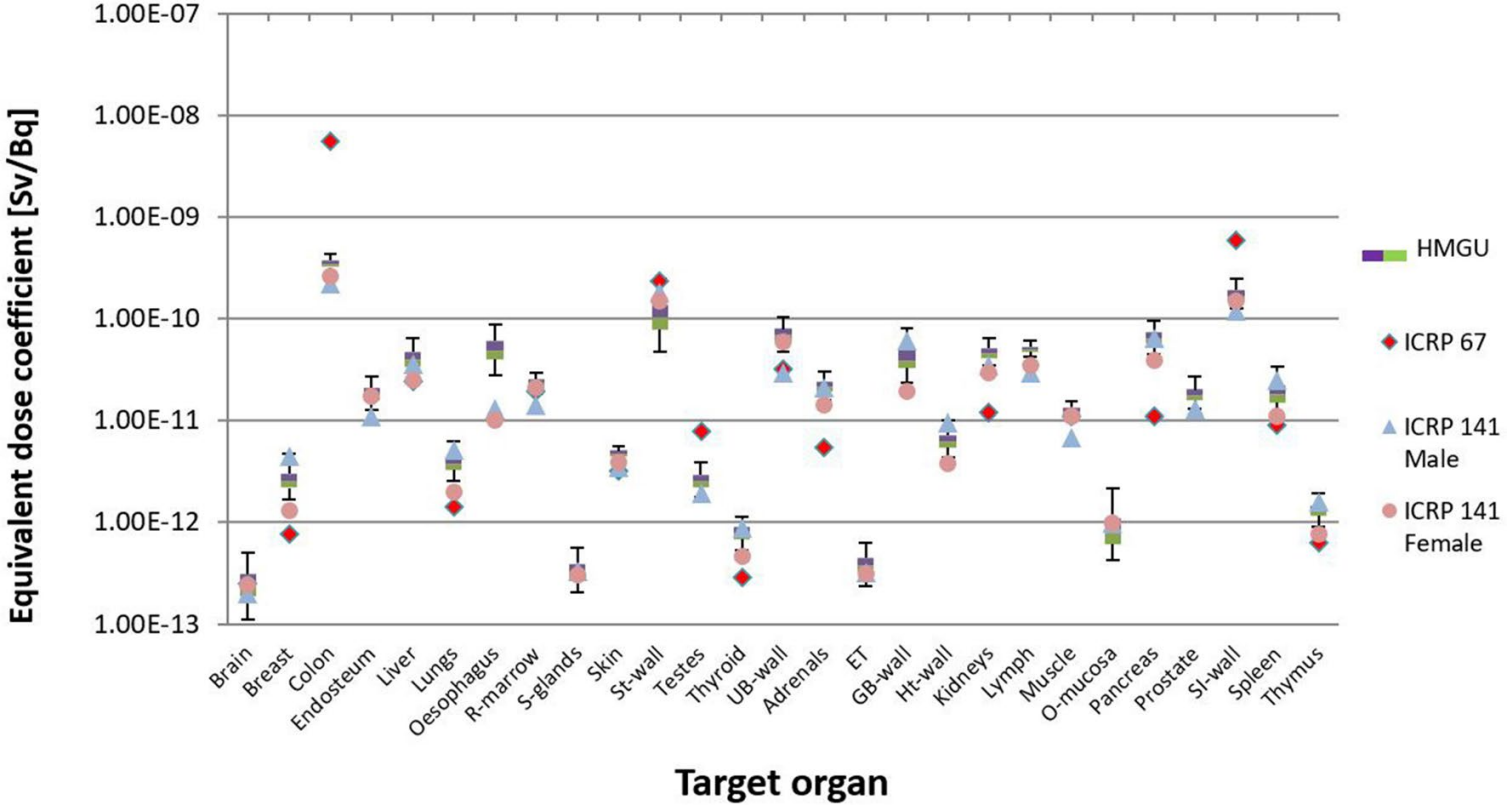
Uncertainty of dose coefficients for ^141^Ce calculated for three phantoms (ICRP reference male, ICRP reference female and Katja). The values of ICRP (1993, 2019) are also shown

**Fig. 10:**
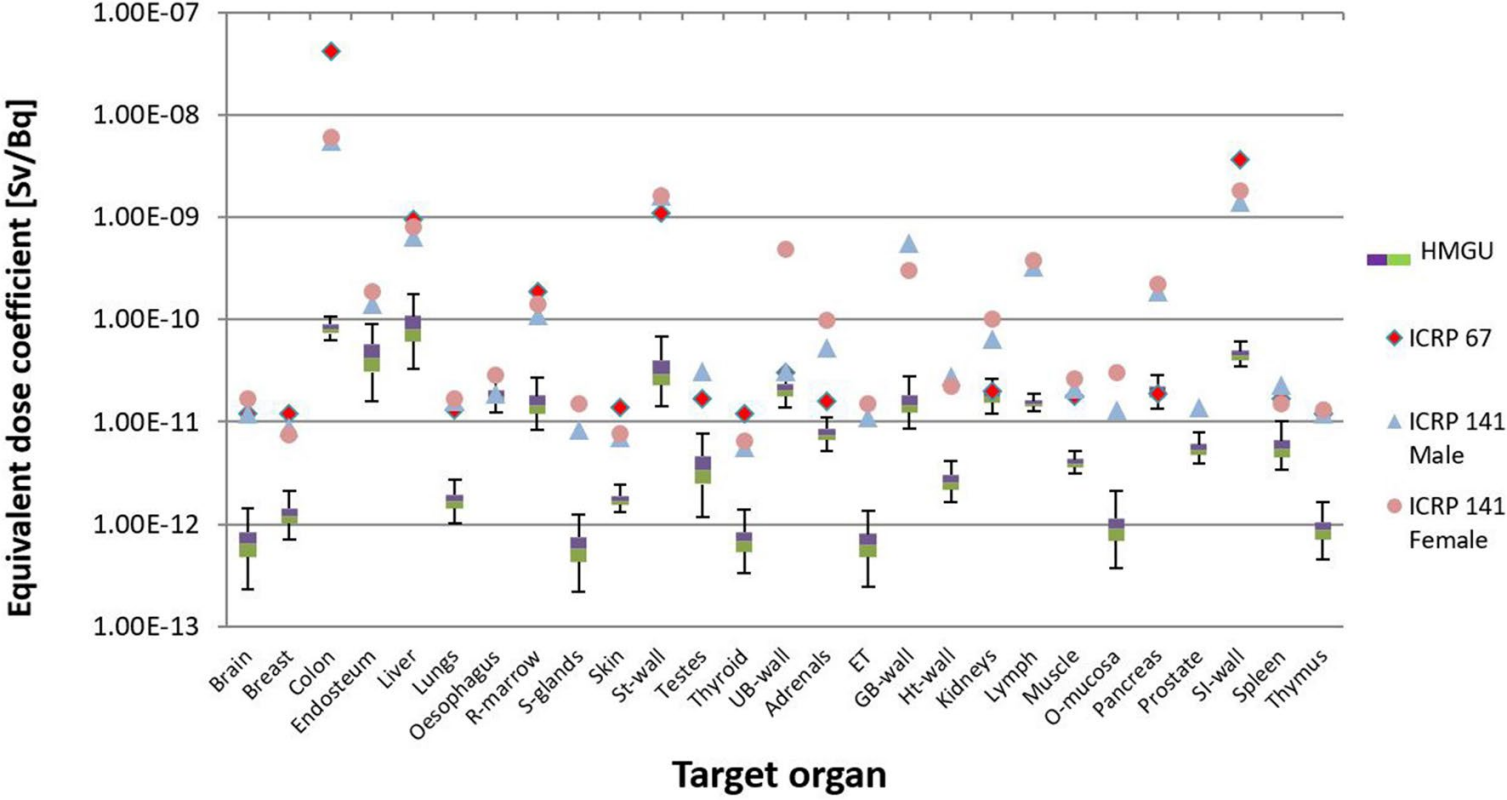
Uncertainty of dose coefficients for ^144^Ce calculated for three phantoms (ICRP reference male, ICRP reference female and Katja). The values of ICRP (1993, 2019) are also shown

The comparison of the calculated values with the reference values from ICRP (ICRP 1993, 2019) is shown in Figs. 9 and 10. Finally, Fig. 12 shows the comparison of the calculated detriment weighted dose coefficient for ^141^Ce and ^144^Ce with the reference values from ICRP (ICRP 1993, 2019), while Fig. 12 shows the same for the calculation with three phantoms.

## Discussion

Uncertainties in absorbed dose involve many different sources. However, in the present study mainly those attributed to the uncertainties in the biokinetic model parameters and the uncertainties of the *S* values were investigated.

As mentioned above, the calculated uncertainties for the *S* values were obtained from the values calculated using seven different voxel phantoms. These phantoms differ not only in body size but also in the organs that have been segmented. In some cases, dose coefficients for organs that have not been segmented in a phantom were replaced by those for existing “surrogate” organs located in an approximately similar location in the body.

The uncertainty of the biokinetic parameters mostly results from the assumption that the coefficient of variation $c_{{\text{v}}}$ is 20%. With this assumption, one achieves a narrow uncertainty range for the parameters of 15.91–31.64%. Compared with this range, the uncertainty ranges from 0.16 to 32.26% and from 0.29 to 32.18% of the normally distributed *S* values for all phantoms (Table 4) and for three phantoms (Table 5), respectively, is larger. However, the difference between the maxima of the coefficient of variation for biokinetic parameters and *S* values is small.

The choice of a $c_{{\text{v}}}$ value of 20% meant that the coverage probability covered the assumed nominal confidence level of 95%. In this case, the coverage probability is greater than the nominal coverage probability, i.e. the assumption of $c_{{\text{v}}}$ = 20% for a confidence interval of 95% is "conservative". This can increase the actual uncertainty. However, since no data was available, this seemed the only reasonable assumption*.*

The uncertainty for log-normally distributed *S* values, calculated for all phantoms, is very large. For example, for ^141^Ce and target organ “extrathoracic airways” the geometric standard deviation reached a maximum value of 9.11; for ^144^Ce and target organ “oral mucosa” the geometric standard deviation reached a maximum value of 7.31; for ^144^Pr and target organ “lymph” the geometric standard deviation reached a maximum value of 14.03; and for ^144m^Pr and target organ “stomach wall” the geometric standard deviation reached a maximum value of 8.81.

When using the three phantoms for which the dosimetric methods used were the same, the geometric standard deviation of *S* values for ^141^Ce was reduced to a maximum value of 4.15 for the target organ “oral mucosa”; for ^144^Ce and target organ “oral mucosa” the geometric standard deviation reached a maximum value of 7.31, which is the same as calculated for all phantoms; for ^144^Pr and target organ “salivary glands” the geometric standard deviation reached a maximum value of 12.00; and for ^144m^Pr and target organ “oesophagus” the geometric standard deviation reached a maximum value of 5.75.

For all seven phantoms, the highest UFs for dose coefficients for ^141^Ce and ^144^Ce were obtained for the target organs “stomach wall”, “colon”, and “small intestine wall”, with values of 5.06, 3.08, and 2.92 for ^141^Ce, and 7.38, 4.62 and 3.81 for ^144^Ce, respectively. But also for the target organs, “oral mucosa” for both radio-isotopes and “brain”, “endosteum”, “liver”, “salivary glands”, “testes”, “thyroid” and “extrathoracic airways” for ^142^Ce, UF values were rather high and greater than 2.

When calculating with three phantoms, the highest UFs for dose coefficients were significantly smaller. For ^141^Ce these were observed for the target organs “stomach wall”, “oral mucosa” and “brain” with values of 2.27, 2.26, and 2.13, respectively. All other target organs showed values of less than 2. For ^144^Ce, the target organs “testes” and “brain” had the highest UFs with values of 2.55 and 2.48, respectively.

Because the deviations in calculated *S* values between different voxel phantoms combined with the uncertainties of biokinetic parameters were rather high, the corresponding uncertainties of calculated dose coefficients were also high.

The UFs of detriment-weighted dose coefficients were lower in comparison to those of equivalent dose coefficients, because of the weighted averaging applied in the calculation of detriment-weighted dose coefficients.

When all phantoms were considered, the UFs were 2.54 and 3.90 for ^141^Ce and ^144^Ce, respectively. In contrast, when only the three phantoms were used the values were significantly lower, i.e., 1.26 and 1.31 for ^141^Ce and ^144^Ce, respectively.

Comparison of the UFs shown in Tables 6 and 7 demonstrates the great influence of the uncertainties in the *S* values on the uncertainties of dose coefficients.

As already mentioned earlier, reference values published in ICRP Publication 67 (ICRP 1993) and in ICRP Publication 141 (ICRP 2019) were also used to compare with the equivalent and detriment-weighted dose coefficients calculated in the present work. For this comparison, the values from ICRP Publication 141 were given separately for males and females. This comparison demonstrated the development in biokinetic and dosimetric models and voxel phantoms in the internal dose calculation. When all phantoms were used in the calculations for ^141^Ce for most target organs, the calculated values were above the reference values of ICRP Publication 67, but in good agreement with the reference values from ICRP Publication 141 (Fig. 7). For the target organs “brain”, “red bone marrow”, “stomach wall”, “urinary bladder wall”, “muscle”, and “small intestine wall”, the reference values from ICRP Publication 67 were also in good agreement with the calculated values. For ^144^Ce for most target organs, the calculated values were below the reference values (Fig. 8). It was only for the target organs “oesophagus”, “urinary bladder wall”, “kidneys”, and “pancreas”, where the reference values were within the given uncertainty range.

Similarly, when only the three phantoms were used, values calculated for ^141^Ce and most target organs were found to be above the reference values from ICRP Publication 67, but in good agreement with those from ICRP Publication 141 (Fig. 9). Only for target organs “colon”, “testes”, and “small intestine wall”, the reference values of ICRP Publication 67 were above the calculated values, and only for “oesophagus” the reference value of ICRP Publication 141 was outside the calculated uncertainty range. It should be noted that ICRP Publication 67 does not provide explicit values for colon, but for Upper Large Intestine (ULI) and Lower Large Intestine (LLI). Consequently, the values for colon and both isotopes were calculated as mass average according to the formula 0.57 × ULI + 0.43 × LLI (ICRP 1998). For the target organs “brain”, “liver”, “red bone marrow”, “stomach wall”, and “muscle”, the reference values from ICRP Publication 67 were in good agreement with the calculated values.

For ^144^Ce, when three phantoms were used the situation was the same as all phantoms were used (Fig. 10). For most target organs, the calculated values were below the reference values. Only for the target organs “oesophagus”, “urinary bladder wall”, “kidneys”, and “pancreas”, the reference values were within the given uncertainty range.

For the calculation of the dose coefficients of ICRP Publication 67 (ICRP 1993) a mathematical hermaphrodite phantom was used that is different from the voxel phantoms used in the present study. For some organ source-target pairs, the *S* values derived from the mathematical phantom differed greatly from the respective *S* values derived from the ICRP reference voxel phantoms used in the present calculations. In addition, in the mathematical phantom, the *S* values for electrons were not explicitly simulated but approximated as in Eq. 5 (ICRP 1979). As previous studies have shown, using different formats of phantoms, i.e. mathematical or voxel phantoms, can cause large differences in the dose calculation for some organs and radionuclides. Probably, for this reason, the equivalent dose coefficients from ICRP Publication 67 were often not within the calculated uncertainty range.

**Fig. 11:**
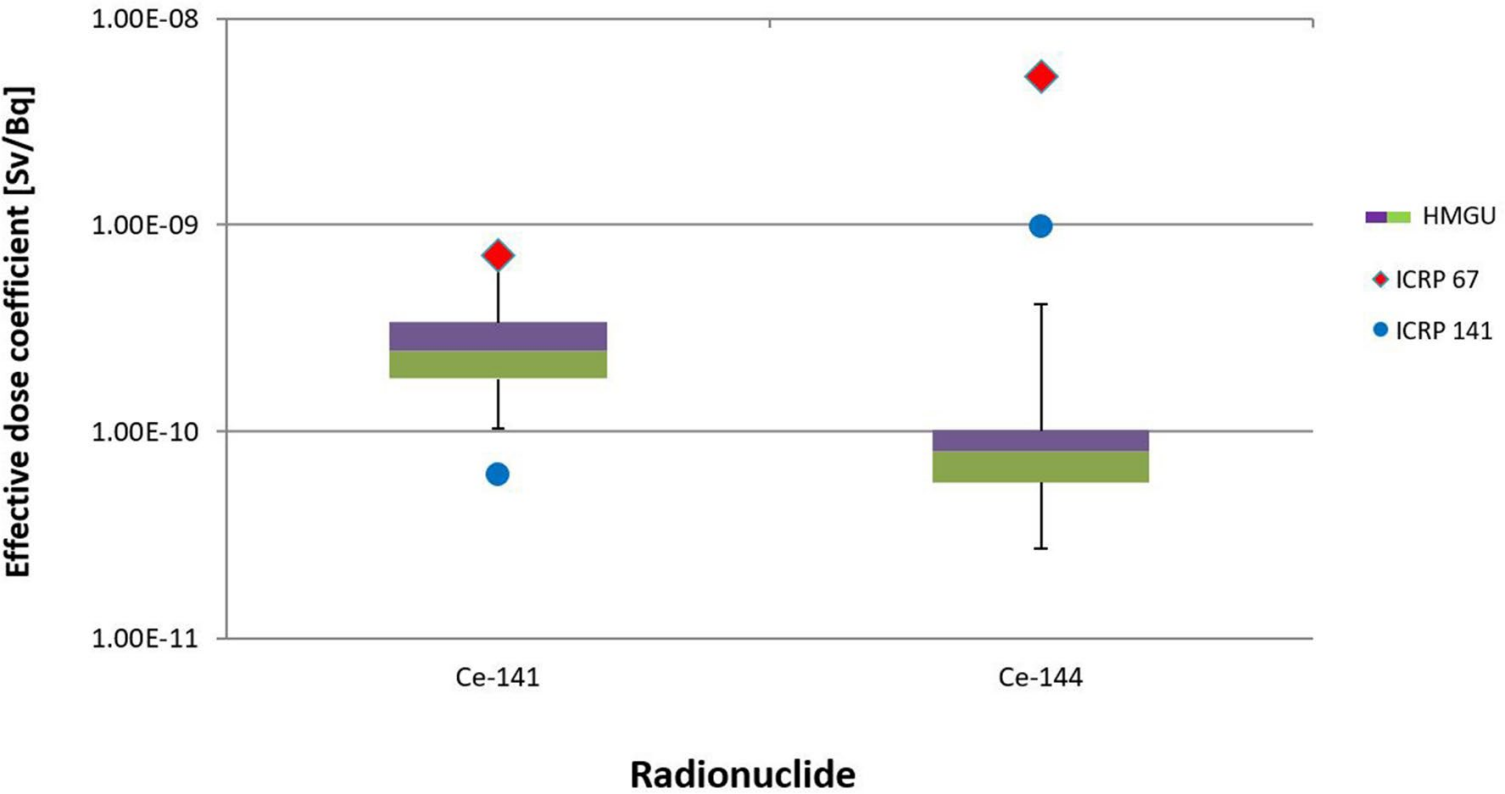
Uncertainty of detriment weighted dose coefficients for ^141^Ce and ^144^Ce, calculated for the seven phantoms of Table 1, together with the values of ICRP (1993, 2019)

When all phantoms were used, the detriment-weighted dose coefficient calculated for ^141^Ce was in good agreement with the reference value of ICRP Publication 67, but the reference value from ICRP Publication 141 was outside of the present uncertainty range. For ^144^Ce, however, both reference values were higher than the calculated values and lay outside the uncertainty range (Fig. 11).

**Fig. 12:**
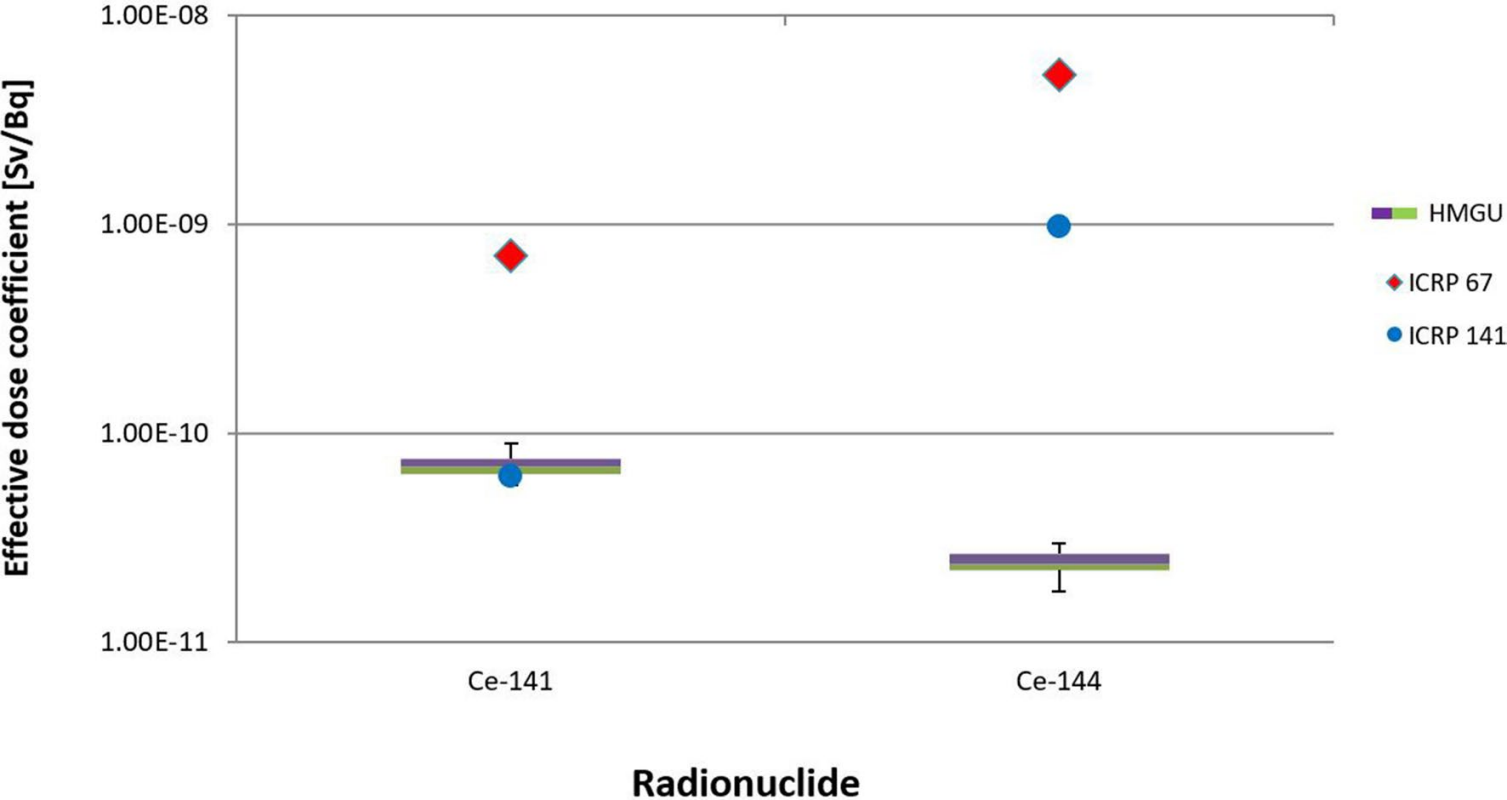
Uncertainty of detriment weighted dose coefficients for ^141^Ce and ^144^Ce, calculated for three phantoms (ICRP reference male, ICRP reference female and Katja, together with the values of ICRP 1993, 2019)

When the three phantoms were used in the calculations, for both ^141^Ce and ^144^Ce the reference values from ICRP Publication 67 were higher than the calculated values and lay outside the present uncertainty range. However, for ^141^Ce the reference value from ICRP Publication 141 lay within the uncertainty range (Fig. 12).

It should be noted that for the present study the tissue-weighting factors of ICRP Publication 103 (ICRP 2007) were used. However, the effective dose coefficients reported by ICRP (ICRP 1993) were calculated with the tissue-weighting factors of ICRP Publication 60 (ICRP 1991). This is one of the reasons why the reference values from ICRP Publication 67 and our calculated values are so different.

In general, overall uncertainties in dose coefficients can be attributed to uncertainties related to biokinetic model parameters and *S* values. However, it is not clear why the uncertainties for some organs are bigger than those for other organs. To clarify this, sensitivity analyses are required.

It is noted that in the calculation of the uncertainty of detriment-weighted dose coefficients any uncertainty in tissue-weighting factors and in radiation-weighting factors was not considered in the present study. In a full assessment of uncertainties, this source of uncertainty should also be considered. This was, however, beyond the scope of the present study.

## Conclusion

In the present study, a method was developed that allowed calculation of uncertainties in equivalent and detriment-weighted dose coefficients of lanthanides, in particular for ingestion of ^141^Ce and ^144^Ce.

The uncertainty factors for equivalent dose coefficients estimated based on seven different voxel phantoms of different stature and sex were found to be in the range of 1.20–7.38, while those for detriment-weighted dose coefficients were found to be 2.54 and 3.90 for ^141^Ce and ^144^Ce, respectively. These differences were attributed to the different methodologies used in calculating uncertainty factors, including different anthropomorphic phantoms, anatomical differences among the individuals whose medical data were used for the construction of the phantoms, differences in electron transport calculations, differences in skeletal dosimetry, etc.

When those three phantoms were used for which the most similar methodologies were applied, substantially smaller uncertainties of dose coefficients were observed. In this case, the uncertainty factors for equivalent doses were in the range from 1.18 to 2.55, while those for detriment-weighted dose coefficients were 1.26 and 1.31 for ^141^Ce and ^144^Ce, respectively. This means that if similar dosimetric methodologies are applied, the uncertainties are reduced.

Moreover, it was also found that the equivalent and detriment-weighted dose coefficients for the radionuclides considered mostly differ from the reference values reported by ICRP Publication 67. As mentioned above, this can also be explained by different methodologies used to construct the anthropomorphic phantoms considered, anatomical differences among individuals, differences in electron transport, and differences in calculating effective dose.

The uncertainty analysis presented here can further be used in a sensitivity analysis to identify the influence of the model parameters involved in the dose calculation. As a result, insensitive parameters can be identified and fixed, to reduce model complexity and thus required computing time. In contrast, one can concentrate research on the most influential parameters and investigate these in more detail, to improve the biokinetic models and, consequently, to reduce the uncertainty of dose coefficients.
